# Concurrent Tuberculous Optic Neuritis and Optic Perineuritis in a Patient With Human Immunodeficiency Virus (HIV)

**DOI:** 10.7759/cureus.55867

**Published:** 2024-03-09

**Authors:** Muhammat Asyari Ismail, Nor Syahira Shariffudin, Nor Fadzillah Bt Abd Jalil, Tze Cheng Yew, Wan-Hazabbah Wan Hitam

**Affiliations:** 1 Department of Ophthalmology and Visual Sciences, School of Medical Sciences, Health Campus, Universiti Sains Malaysia, Kubang Kerian, MYS; 2 Department of Ophthalmology, Hospital Melaka, Melaka, MYS; 3 Department of Radiology, Hospital Melaka, Melaka, MYS

**Keywords:** corticosteroids, human immunodeficiency virus, optic perineuritis, optic neuritis, tuberculosis

## Abstract

Concurrent tuberculous optic neuritis (ON) and optic perineuritis (OPN) in a patient with human immunodeficiency virus (HIV) is extremely rare. HIV-induced progressive CD4 depletion is associated with an increased risk of tuberculosis (TB), disseminated TB, and death. Early detection and initiation of anti-TB therapy with corticosteroid commencement helps in achieving better visual outcomes. Interestingly, we report a case of concurrent ON and OPN in a patient with HIV-TB co-infection. A 29-year-old lady, a prisoner, with newly diagnosed treatment-naive HIV, presented with acute-onset reduced vision in the left eye for 10 days. It was associated with pain in eye movement and headache. The patient was known to be a drug abuser since the age of 19 years and was a sexual worker. Her CD4 count was 292 cells/mm^3^.Visual acuity of the right eye was 6/12 with a pinhole of 6/9, and there was no perception of light (NPL) in all four quadrants of the left eye. Relative afferent pupillary defect (RAPD) was positive in the left eye. Both anterior segments were unremarkable. The right eye fundoscopy showed a normal optic disc, while the left eye showed a hyperemic disc. During subsequent follow-up, the patient had reduced right eye vision, and the vision dropped to 6/30 with a pinhole of 6/15. Her erythrocyte sedimentation rate (ESR) was raised to 88 mm/h. The Mantoux test was positive. Chest radiography was normal. MRI of the brain and orbit showed significant enhancement of the right optic nerve and left optic nerve sheath suggesting the diagnosis of right eye ON and left eye OPN secondary to TB. The patient was co-managed with an infectious disease team. She was started on highly active antiretroviral therapy (HAART) treatment (oral Tenvir-EM and efavirenz) upon presentation. Anti-TB therapy was commenced two months later. She was started on the intensive phase of the anti-TB regime followed by the maintenance phase. Oral dexamethasone was given concurrently according to the central nervous system (CNS) TB regime for six weeks. During follow-up, her right eye visual acuity was 6/9, and her left eye visual acuity improved to 6/12. Fundoscopy showed bilateral pale discs. To date, no episodes of recurrence have been seen.

## Introduction

Concurrent optic neuritis (ON) with optic perineuritis (OPN) is an uncommon but severe consequence of tuberculosis (TB) infection. It is caused by the *Mycobacterium tuberculosis* bacteria, which can spread from the lungs to other parts of the body including the optic nerve and optic nerve sheath. The condition is significantly more common in people with weakened immune systems [1-3], and early detection and treatment are critical in preventing permanent optic nerve damage and preserving eyesight [3-5]. Diagnosis of concurrent ON with OPN involves a combination of clinical observations, laboratory tests, and imaging studies. Concurrent ON with OPN secondary to TB is a very rare but serious condition that can result in permanent vision loss if left untreated. With early diagnosis and prompt treatment, prognosis is generally good, and most patients recover their vision [3-5]. In this paper, we report on a very rare case of concurrent TB ON and OPN in a patient newly diagnosed with human immunodeficiency virus (HIV).

## Case presentation

A 29-year-old female convict with newly diagnosed treatment-naive HIV presented with acute-onset reduced vision in the left eye (LE) for 10 days. It was associated with pain with eye movement and headache. The patient had a history of substance abuse since the age of 19. She was also engaged as a commercial sex worker. The patient was diagnosed with HIV in 2022 with a baseline CD4 count of 292 cells/mm^3^. She did not show symptoms of TB, which include prolonged cough, fever, night sweats, loss of weight, and loss of appetite. The patient had no history of contact with TB patients.

**Figure 1:**
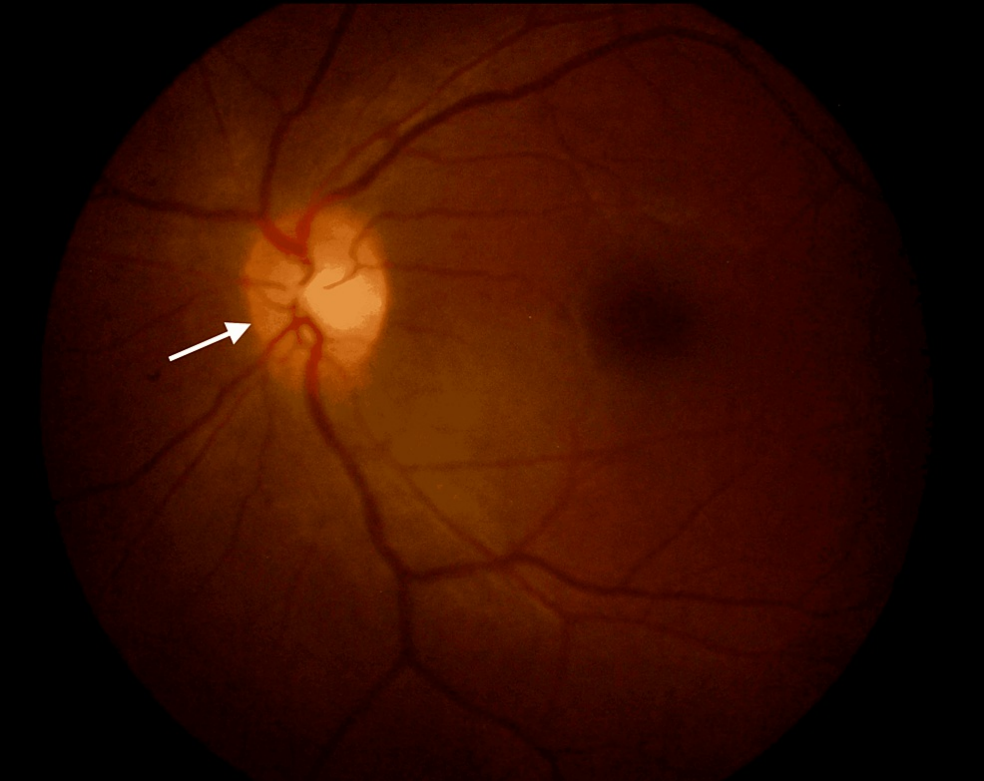
Left eye hyperemic optic disc during the initial presentation The white arrow shows the left eye hyperemic optic disc.

On presentation, her visual acuity in the right eye (RE) was 6/12 with a pinhole of 6/9, and there was no perception of light (NPL) in the LE. Left relative afferent pupillary defect (RAPD) was positive. A fundoscopy of the LE showed a hyperemic disc (Figure 1), while the RE was normal.

There was no sign of retinitis or vasculitis. The physical examination was unremarkable; there was no lymphadenopathy or organomegaly. Respiratory and neurological examinations were also normal. Based on clinical findings of poor visual acuity and hyperemic LE optic disc, we suspected the patient to have ON. She was planned to do an MRI on the follow-up appointment. 

**Figure 2:**
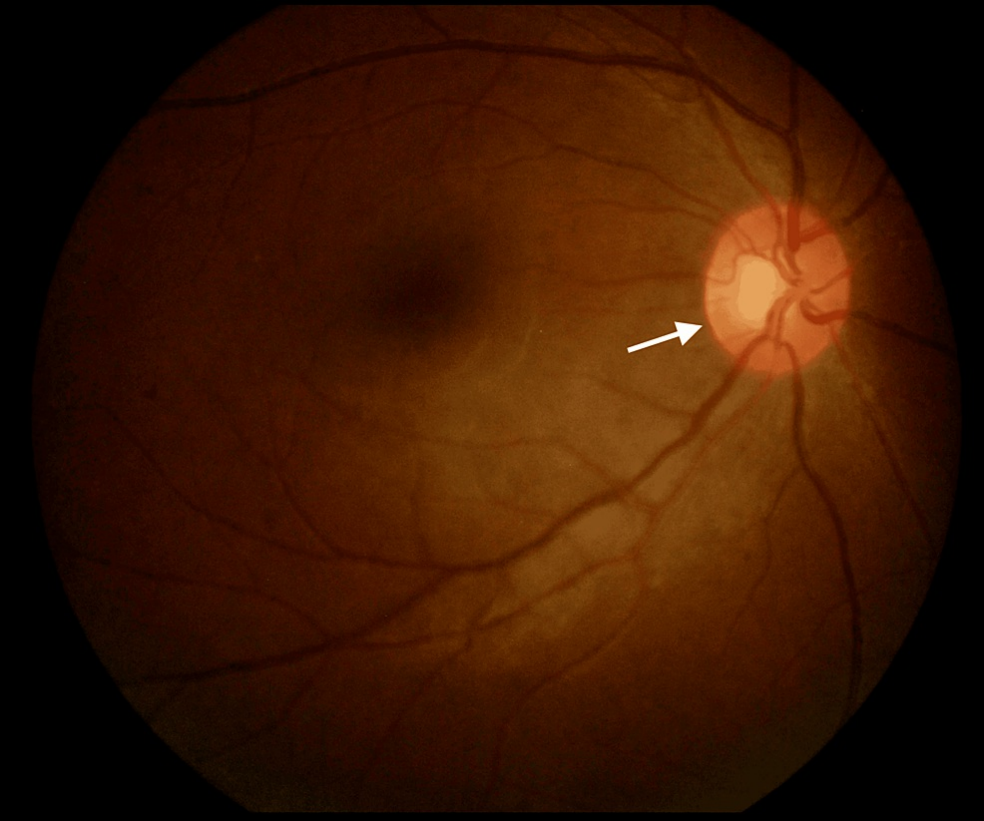
Right eye hyperemic optic disc on the subsequent follow-up The white arrow shows the right eye hyperemic optic disc.

On follow-up, her RE vision further deteriorated. Her visual acuity in the RE was 6/30 with a pinhole of 6/15. A fundoscopy of the RE showed a hyperemic disc (Figure 2).

**Figure 3:**
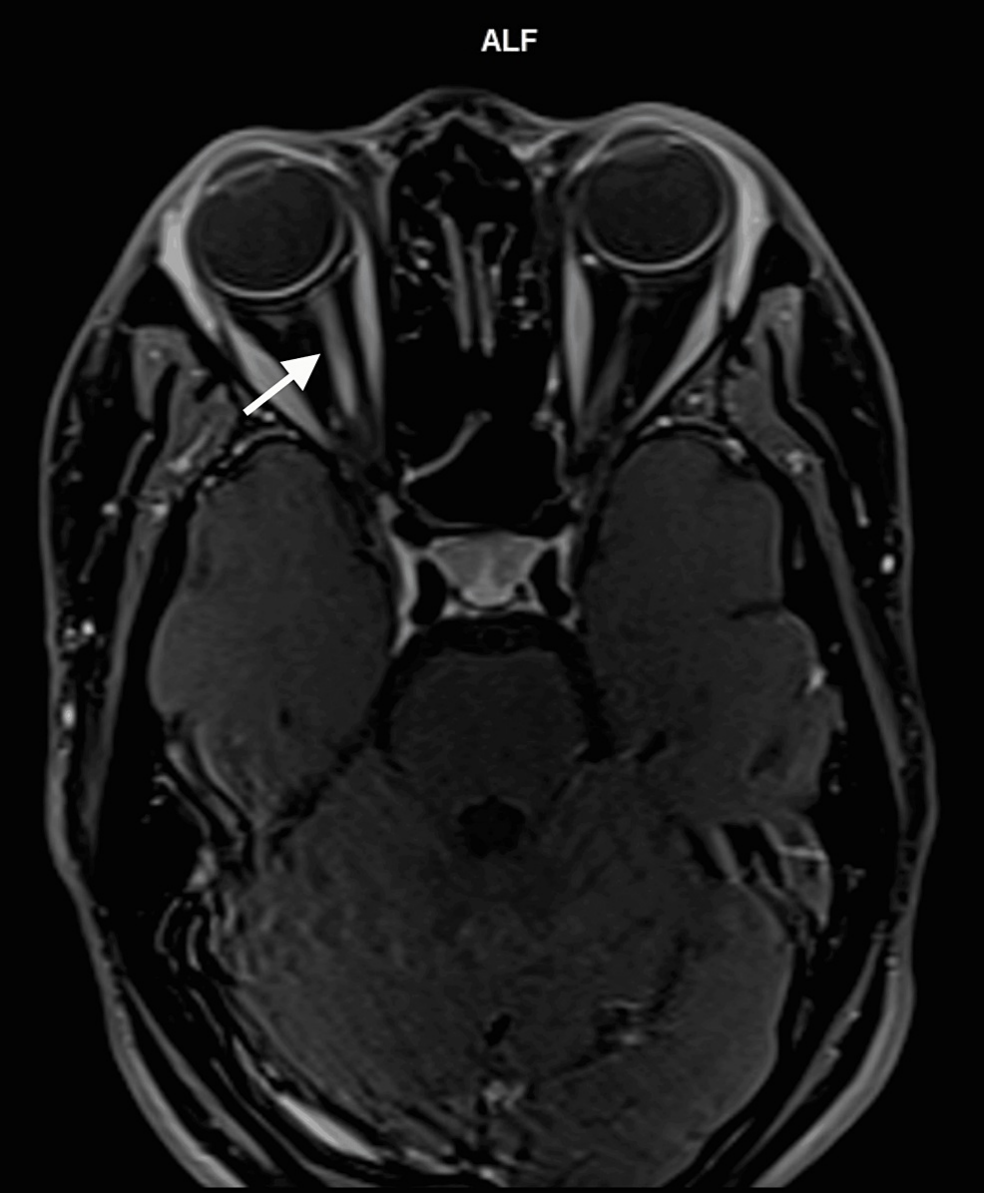
MRI film axial view: right optic nerve enhancement The white arrow shows the hyperintense right intraorbital segment of the right optic nerve.

The patient's erythrocyte sedimentation rate (ESR) was elevated (88 mm/h), and the Mantoux test was positive (21 mm). The chest radiography was normal. Venereal disease research laboratory (VDRL) and toxoplasma serology tests were negative. Antinuclear antibody (ANA) and rheumatoid factor (RF) tests were also negative. An MRI of the brain and orbit showed significant enhancement of the right optic nerve, suggestive of RE ON (Figure 3).

**Figure 4:**
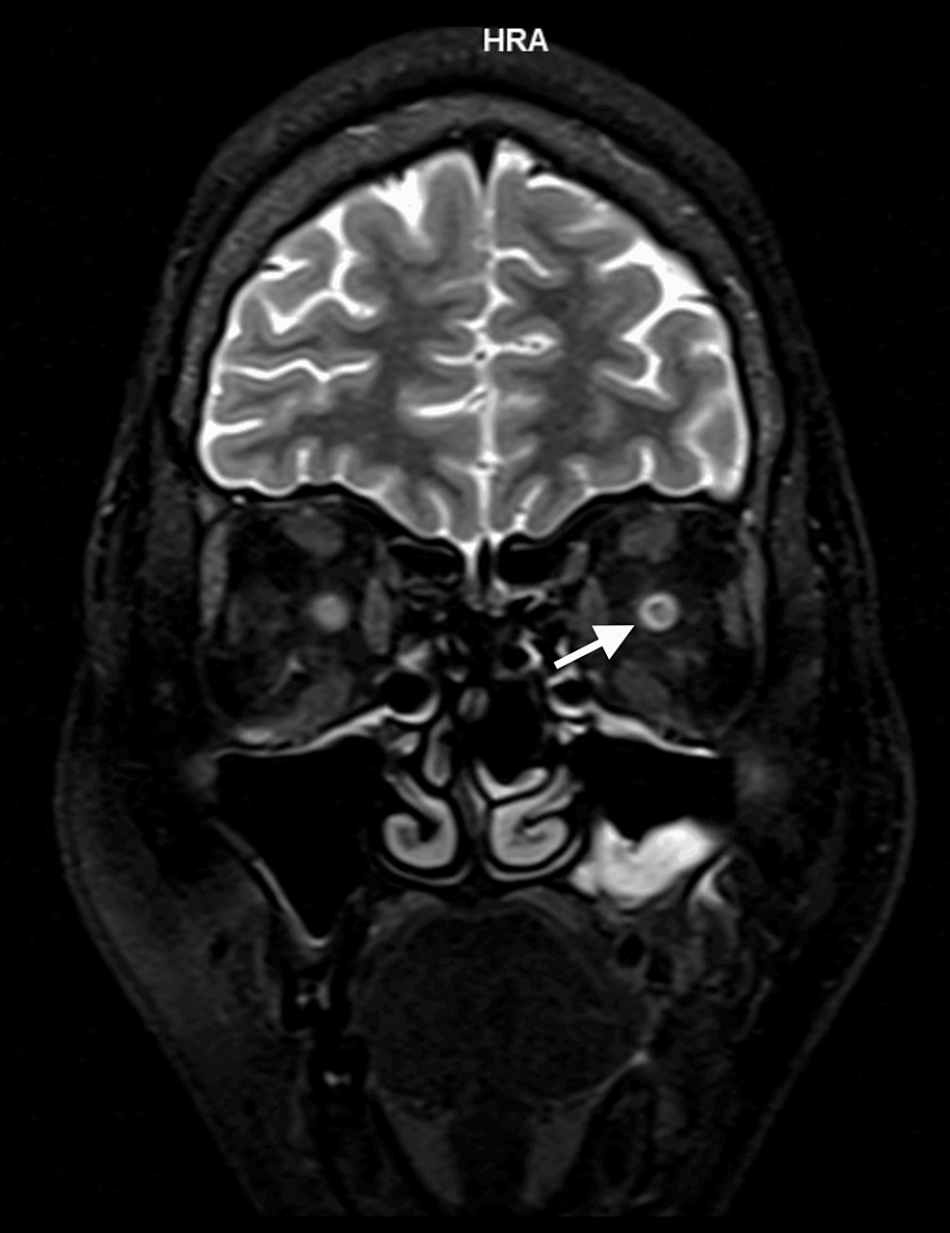
MRI film coronal view: left perineural enhancement ("doughnut sign") The white arrow shows the left eye optic nerve sheath enhancement.

The left optic nerve showed the presence of perineural enhancement ("doughnut" sign), suggestive of LE OPN (Figure 4).

**Figure 5:**
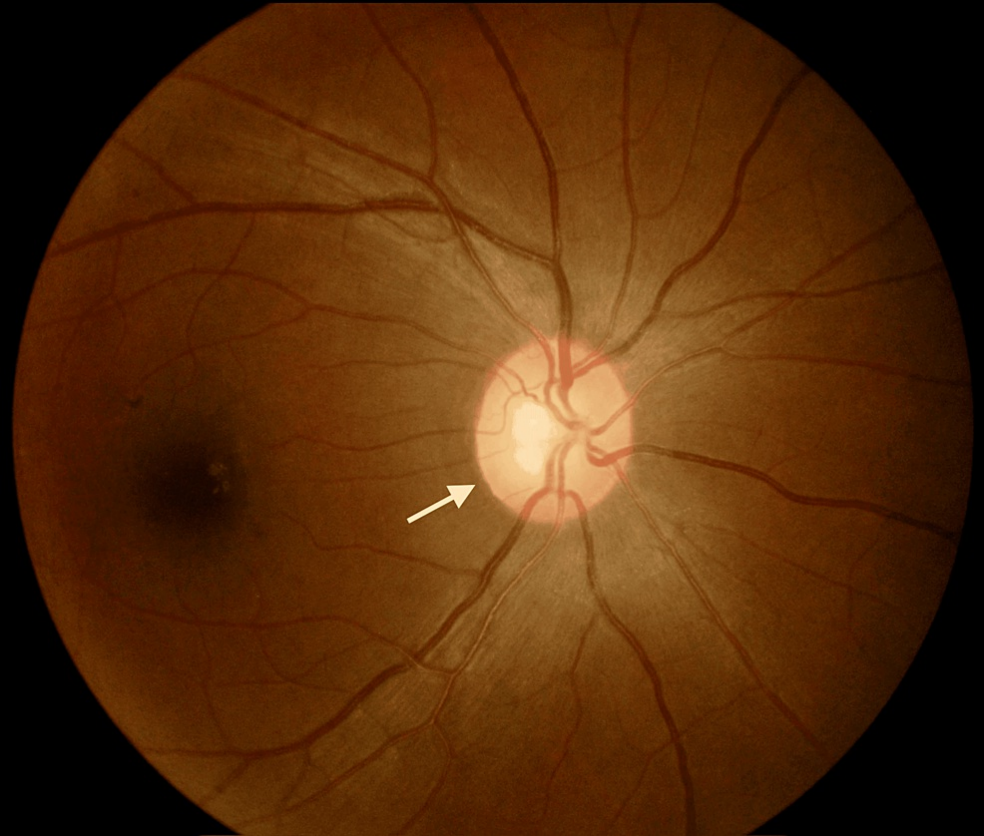
Right eye optic disc atrophy during the six-week follow-up The white arrow shows the right eye optic disc atrophy.

**Figure 6:**
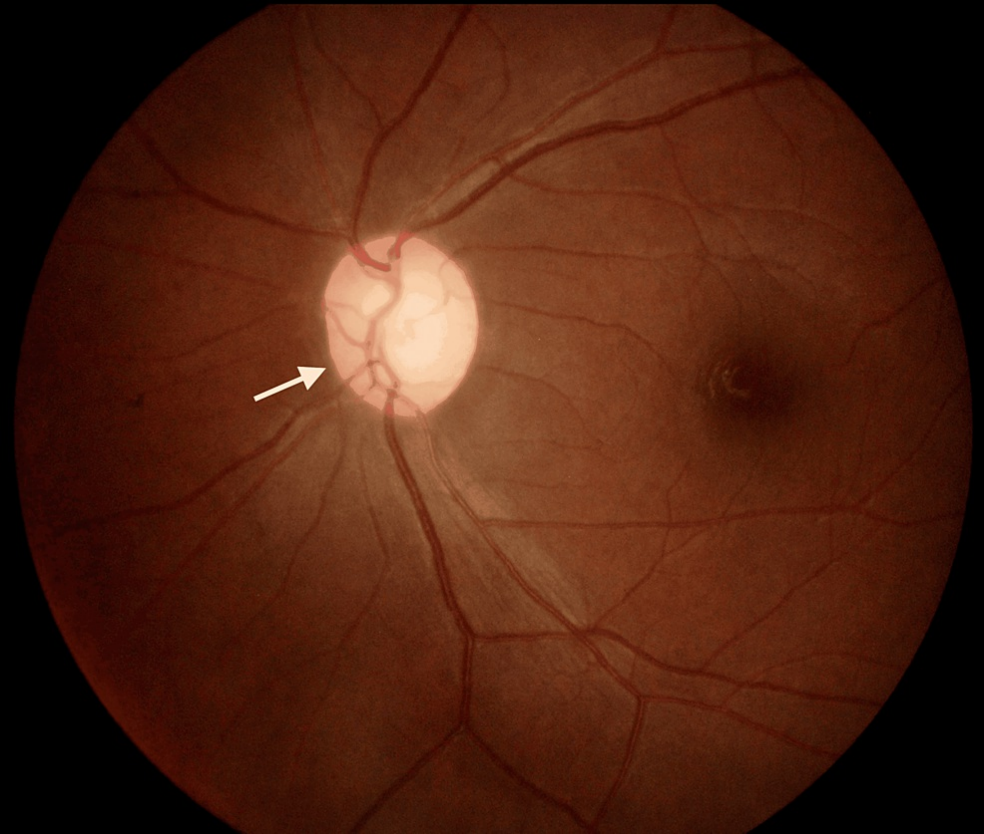
Left eye optic disc atrophy during the six-week follow-up The white arrow shows the left eye optic disc atrophy.

The patient was diagnosed to have concurrent right ON with left OPN secondary to TB. She was co-managed with an infectious disease team. She was started on highly active antiretroviral therapy (HAART) treatment (oral Tenvir-EM and efavirenz) upon presentation. Anti-TB therapy was commenced two months later. She was started on the intensive phase of the anti-TB regime followed by the maintenance phase. Oral dexamethasone was also given concurrently according to the central nervous system (CNS) TB regime for six weeks. During follow-up, her RE visual acuity improved to 6/9, and the LE visual acuity also improved to 6/12. Both optic discs were pale (Figure 5 and Figure 6).

There were no episodes of recurrence during follow-up at six months, with visual acuity in both eyes remaining the same. The anti-TB regime was continued for nine months. Her HIV RNA copies were not detected since February 2023, and her CD4+ count had gradually improved from 164 cells/uL in February 2023 to 522 cells/uL in June 2023. 

## Discussion

In Southeast Asia, TB is extremely common. The region is thought to be responsible for 43% of the world's TB burden, according to the World Health Organization (WHO) [1]. In 2020, the TB incidence in Malaysia was 72.4 out of every 100,000 people, and the incidence of HIV-TB co-infection was 5.2 out of every 100,000 people [2]. The overall incidence of TB among four state prisons with a high burden of TB in Malaysia was 440/100,000 [6]. Extrapulmonary involvement is observed in more than 50% of patients who have both HIV and TB. The risk of extrapulmonary TB (EPTB) is higher in patients with a low CD4 count; 70% of patients with a CD4 count of less than 100 develop EPTB compared to 28% with a CD4 count of more than 300 [3].

Ocular involvement occurs in about 1-2% of patients with TB. Ocular involvement can result from hematogenous spread, direct local extension, or a hypersensitive response to distant infection [7]. Optic neuropathy may occur during TB infection due to tubercular perineuritis, ON (which can involve, as in our case, both ON and OPN concurrently), papilloedema and consecutive optic atrophy due to raised intracranial pressure, endarteritis of the optic nerve, toxic neuritis due to drugs (ethambutol and isoniazid), arachnoiditis of the optic chiasma, or compression of the optic nerve by tuberculoma [8].

ON refers to inflammation of the optic nerve. It is usually associated with multiple sclerosis, but the causes vary. Depending on the aetiology, visual prognoses and risks of recurrent injury may also vary. Rapid and accurate diagnosis of ON may be critical for limiting vision loss, future neurological disability, and organ damage. Meanwhile, OPN is an orbital inflammatory disease in which the focus of the inflammatory response is the optic nerve sheath. Most cases are isolated and idiopathic, but they can occasionally occur as manifestations of specific infectious or inflammatory diseases [9]. In both ON and OPN, patients typically experience acute monocular visual loss, pain with eye movement, and either a normal or a swollen optic disc [9]. Our patient had an acute visual loss with pain accompanying eye movement and a hyperemic disc upon presentation. There was no immune reconstitution inflammatory syndrome (IRIS) observed. The natural histories and responses to treatment of ON and OPN may differ, however.

The diagnosis of OPN is typically based on a combination of clinical and radiographic findings. The clinical presentation of OPN is similar to that of ON, as noted above. Visual loss usually progresses for several weeks until it is correctly diagnosed and treatment is initiated. On the other hand, in ON, the patient will usually spontaneously recover with or without corticosteroid treatment. One obvious feature that differentiates OPN from ON is that OPN patients will have prompt and dramatic relief of pain and improvement in vision after corticosteroid therapy [9]. However, relapse is common upon tapering or stopping treatment. In addition to visual complaints, OPN can also present with other concurrent symptoms of orbital inflammation, such as diplopia, subtle ptosis, or chemosis.

MRI scans of patients with OPN typically show a characteristic pattern of enhancement around the optic nerve ("tramtrack" in the axial view and "doughnut" in the coronal view). In addition, MRI scans sometimes show inflammatory changes in intraconal orbital fat. In occasional cases of OPN, the substance of the optic nerve also shows enhancement, presumably due to inflammation of the intraneural pial septa as well as the nerve sheath [8]. In contrast, MRI for ON demonstrates contrast enhancement in the optic nerve and multiple hyperintensities of white matter lesions.

In our patient, there were radiological changes in both ON and OPN. Her RE showed optic nerve enhancement on post-contrast MRI, while her LE showed perineural enhancement of the optic nerve: "doughnut" sign in the coronal view. Other investigations that may be useful to rule out infection and inflammatory or autoimmune diseases include serological tests for syphilis, Mantoux tests and chest radiographs for TB, serum angiotensin-converting enzyme for sarcoidosis, and ESR [10].

Treatments for OPN include high doses of systemic corticosteroids for both idiopathic and secondary cases [5,10,11]. In cases of refractory OPN, there are also reports of treatment with radiation therapy and immunosuppressants. In contrast, systemic corticosteroids are used to hasten visual recovery in ON cases. One study showed that corticosteroids do not influence visual outcomes in patients in ON; therefore, many patients are managed expectantly (i.e., without treatment) [9].

Our patient was treated with oral corticosteroids based on the TB meningitis regime after a discussion with the infectious disease team. A lumbar puncture was performed and showed a normal opening pressure and cerebrospinal fluid (CSF) differential. In TB meningitis, a chemocytological pattern with an inflammatory reaction is expected along with increased protein levels, hypoglycorrhachia, and an increased number of lymphocytes, with a predominance of lymphomononuclear cells. The opening pressure is generally high, and the liquid assumes a turbid aspect [12]. However, CSF studies can yield normal results in up to 25% of patients [12].

CSF culture is the gold standard for TB meningitis. People with HIV/acquired immunodeficiency syndrome (AIDS) have a higher culture positivity than uninfected patients, probably due to the higher bacilliferous load during HIV-TB co-infection. The test's sensitivity is around 50-70%. Another test used to diagnose TB meningitis is the GeneXpert MTB molecular test. Initial studies have indicated a sensitivity of 67% in the general population and only 36% in people living with HIV/AIDS (PLWHA), demonstrating that the isolated use of this test does not exclude the disease and other diagnostic methods should be used concurrently [13]. In our case, both the CSF culture for MTB and the CSF GeneXpert MTB test showed negative results. Thus, the patient was presumed to have TB based on ocular findings and a positive Mantoux test.

The influence of HIV infection on intracranial imaging studies of TB meningitis has been examined in several studies. Meningeal enhancement was more common in HIV-infected individuals, and these individuals were also likelier to present with cerebral infarcts and mass lesions and less likely to present with obstructive hydrocephalus [14]. However, none of the findings were observed in our patient. In stage 1 TB meningitis (marked by a GCS of 15, no focal neurological deficits, and an alert and oriented state), the patient was given oral dexamethasone starting with 0.1 mg/kg/day, and the dose was tapered until completed six weeks of treatment. She was also planned for anti-TB treatment according to a TB meningitis regime (two months of ofloxacin, isoniazid, rifampicin, and pyrazinamide and 10 months of isoniazid and rifampicin).

The prognosis of OPN is excellent, provided that patients are detected early and managed correctly. The interval between the onset of visual loss and the initiation of treatment is crucial for determining the visual outcome. One study discovered poor visual outcomes in patients who received delayed treatment (more than one month after visual symptoms began) [9]. The other prognostic factor is the frequency of recurrent attacks. Initiating treatment with a higher dose of corticosteroids (e.g., prednisolone, 80 mg/day) and more prolonged treatment may lessen the likelihood of recurrence at this level [8]. The overall outcome was good for our patient, as her visual acuity in the LE significantly improved from NPL to 6/12 and she had stable visual acuity (6/9) in the RE throughout the disease course. No episodes of recurrence were observed within six months of follow-up. The early initiation of corticosteroids and treatment with anti-TB treatment help in treating OPN as well as ON.

## Conclusions

This case represents an unusual ocular involvement of TB due to the presence of both ON and OPN in the same patient. A high index of suspicion of TB infection in immunocompromised patients helps in early diagnosis and prompt treatment, which may lead to a better visual prognosis. MRI utilization is crucial for accurate diagnosis, facilitating timely intervention and better outcomes in managing TB-related optic neuropathies.
